# Cytokine profile in plasma of severe COVID-19 does not differ from ARDS and sepsis

**DOI:** 10.1172/jci.insight.140289

**Published:** 2020-09-03

**Authors:** Jennifer G. Wilson, Laura J. Simpson, Anne-Maud Ferreira, Arjun Rustagi, Jonasel Roque, Adijat Asuni, Thanmayi Ranganath, Philip M. Grant, Aruna Subramanian, Yael Rosenberg-Hasson, Holden T. Maecker, Susan P. Holmes, Joseph E. Levitt, Catherine A. Blish, Angela J. Rogers

**Affiliations:** 1 Department of Emergency Medicine and; 2 Department of Medicine, Stanford University School of Medicine, Stanford, California, USA.; 3 Department of Statistics, Stanford University, California, USA.; 4 Institute for Immunity, Transplantation, and Infection, Stanford University School of Medicine, Stanford, California, USA.; 5 Chan Zuckerberg Biohub, San Francisco, California, USA.

**Keywords:** COVID-19, Inflammation, Cytokines

## Abstract

**BACKGROUND:**

Elevated levels of inflammatory cytokines have been associated with poor outcomes among COVID-19 patients. It is unknown, however, how these levels compare with those observed in critically ill patients with acute respiratory distress syndrome (ARDS) or sepsis due to other causes.

**METHODS:**

We used a Luminex assay to determine expression of 76 cytokines from plasma of hospitalized COVID-19 patients and banked plasma samples from ARDS and sepsis patients. Our analysis focused on detecting statistical differences in levels of 6 cytokines associated with cytokine storm (IL-1β, IL-1RA, IL-6, IL-8, IL-18, and TNF-α) between patients with moderate COVID-19, severe COVID-19, and ARDS or sepsis.

**RESULTS:**

Fifteen hospitalized COVID-19 patients, 9 of whom were critically ill, were compared with critically ill patients with ARDS (*n* = 12) or sepsis (*n* = 16). There were no statistically significant differences in baseline levels of IL-1β, IL-1RA, IL-6, IL-8, IL-18, and TNF-α between patients with COVID-19 and critically ill controls with ARDS or sepsis.

**CONCLUSION:**

Levels of inflammatory cytokines were not higher in severe COVID-19 patients than in moderate COVID-19 or critically ill patients with ARDS or sepsis in this small cohort. Broad use of immunosuppressive therapies in ARDS has failed in numerous Phase 3 studies; use of these therapies in unselected patients with COVID-19 may be unwarranted.

**FUNDING:**

Funding was received from NHLBI K23 HL125663 (AJR); The Bill and Melinda Gates Foundation OPP1113682 (AJR and CAB); Burroughs Wellcome Fund Investigators in the Pathogenesis of Infectious Diseases #1016687 NIH/NIAID U19AI057229-16; Stanford Maternal Child Health Research Institute; and Chan Zuckerberg Biohub (CAB).

## Abstract 2

The levels of inflammatory cytokines in COVID-19 patients are compared to patients with other critical illness, such as sepsis or acute respiratory distress syndrome.

## Introduction

Numerous studies have established that levels of inflammatory biomarkers are elevated in COVID-19 patients, and higher levels of inflammatory cytokines are consistently associated with more severe disease and worse outcomes (1–4). These data have sparked interest in “cytokine storm” as a major driver of illness severity in COVID-19, and multiple clinical trials are underway to test the efficacy of immunosuppressive therapies, including IL-6 antagonists (5). While it is clear that COVID-19 is a highly inflamed state (similar to other viral respiratory infections including H5N1 influenza, SARS-CoV, and MERS-CoV) (6–8), it is unknown if inflammatory cytokine levels differ in patients with severe COVID-19 compared with critically ill patients with sepsis or acute respiratory distress syndrome (ARDS) due to other causes.

This is an important comparison, as large-scale randomized controlled trials of immune modulators (e.g., anti–IL-1β , activated protein C, steroids) have failed to demonstrate clear benefit in ARDS and sepsis (9). If the inflammatory response in severe COVID-19 is no different than that in ARDS or sepsis, testing of immune modulators in unselected COVID-19 patients may likewise fail, or lead to inconclusive or contradictory results. A better understanding of how the inflammatory state in severe COVID-19 compares with that of other critical illnesses would not only provide insight into pathophysiology, but could inform study design for trials of immunosuppressive treatments for COVID-19 going forward.

Given potential variability in cytokine quantification across platforms, we used a highly standardized Luminex assay to measure plasma levels of 76 cytokines, including 6 inflammatory cytokines associated with cytokine storm (IL-1β, IL-1RA, IL-6, IL-8, IL-18, and TNF-α). We recruited a prospective cohort of patients hospitalized with COVID-19 and compared the cytokine profiles with those from ARDS and sepsis patients enrolled in the Stanford ICU Biobank prior to the COVID-19 pandemic. While our analysis primarily focused on “cytokine storm” cytokines, we also performed a more exploratory analysis of the additional 70 cytokines measured to contribute to a broader understanding of the inflammatory response in moderate and severe COVID-19.

## Results

To compare cytokine expression levels between COVID-19 patients and other critically ill patients, we measured plasma cytokine levels using Luminex in 4 patient groups: moderate COVID-19 (*n* = 6), severe COVID-19 (*n* = 9), ARDS (*n* = 12), and sepsis (*n* = 16). Clinical characteristics of the study participants in each group are shown in Table 1. Of the 9 severe COVID-19 patients, 6 required mechanical ventilation. While only 1 ventilated COVID-19 patient died, 5 had moderate-to-severe ARDS (5) PaO_2_:FIO_2_ [P:F] < 150 at enrollment) and all required prolonged mechanical ventilation (>7 days).

**Figure 1:**
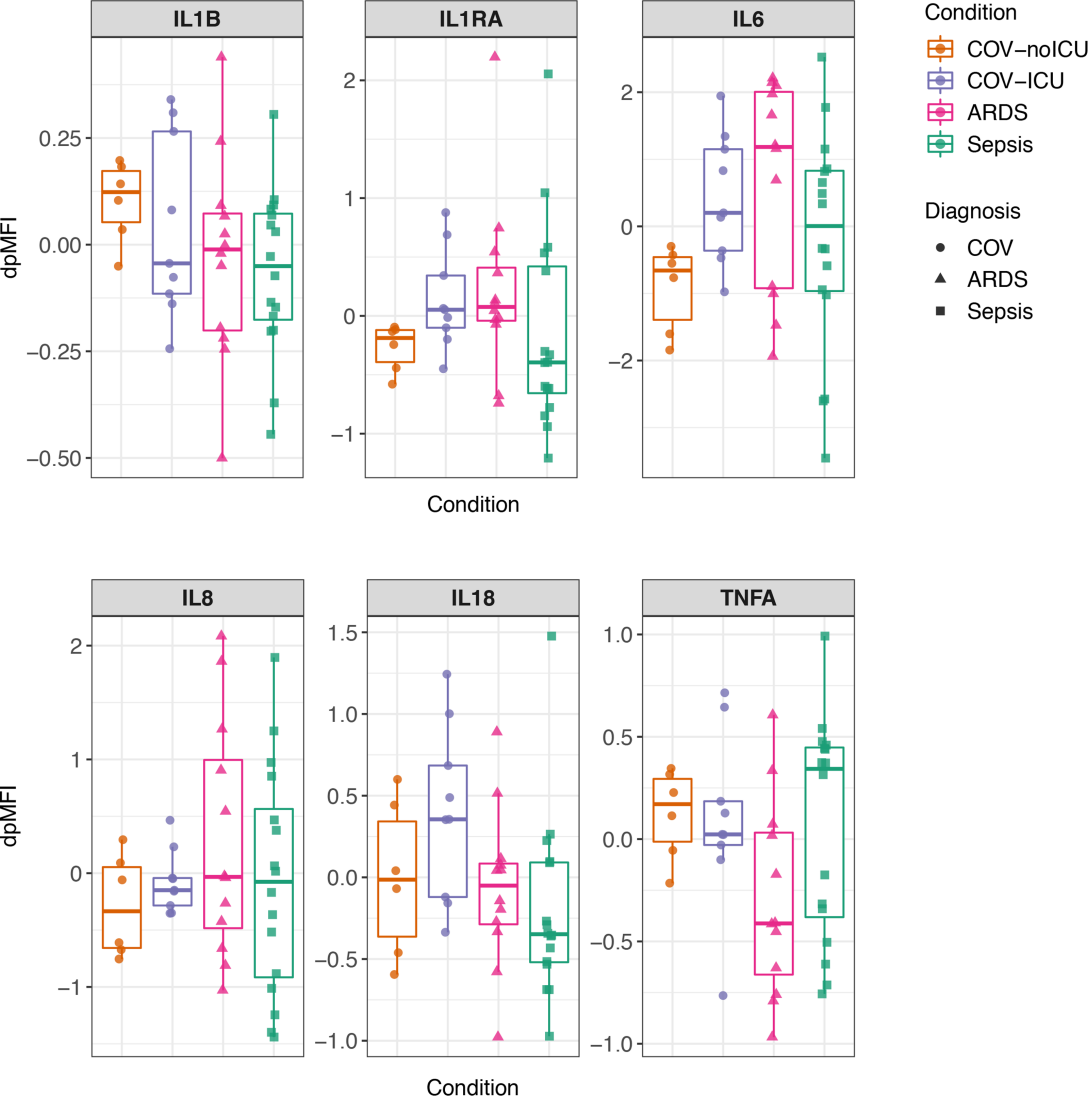
Expression of inflammatory cytokines does not differ between COVID-19 and non-COVID sepsis and ARDS. Cytokine expression levels of 2 replicates per sample were measured in plasma using Luminex and normalized for nonspecific binding, resulting in the mean of plate-detrended median fluorescence intensity (dpMFI) values for each cytokine per sample. Moderate COVID-19 (COV-noICU, orange), *n* = 6. Severe COVID-19 (COV-ICU, purple), *n* = 9. ARDS (pink), *n* = 12. Sepsis (green), *n* = 16. Circles represent subjects with COVID-19, triangles represent subjects with ARDS, and squares represent subjects with sepsis. The box plot visualizes the following summary statistics: the middle line represents the median; the lower hinge corresponds to the first quartile (25th); the upper hinge corresponds to the third quartile (75th); upper and lower whiskers extend from the hinge respectively to the largest value and smallest value or no further than 1.5 × interquartile range; data beyond the whiskers are outliers. Wilcoxon rank sum tests were performed for each cytokine, observing COV-ICU compared with COV-noICU, ARDS, and sepsis. *P* values were adjusted for multiple comparisons using the Benjamini-Hochberg correction method. No cytokines had adjusted *P* < 0.05. *P* values for comparison between COV-noICU and COV-ICU: IL-1β, *P* = 0.53, adjusted *P* = 0.79; IL-1RA, *P* = 0.036, adjusted *P* = 0.22; IL-6, *P* = 0.018, adjusted *P* = 0.16; IL-8, *P* = 0.46, adjusted *P* = 0.79; IL-18, *P* = 0.27, adjusted *P* = 0.70; TNF-α, *P* = 0.86, adjusted *P* = 0.99. *P* values for comparison between COV-ICU and ARDS: IL-1β, *P* = 0.65, adjusted *P* = 0.84; IL-1RA, *P* = 0.92, adjusted *P* = 0.99; IL-6, *P* = 0.51, adjusted *P* = 0.79; IL-8, *P* = 0.60, adjusted *P* = 0.83; IL-18, *P* = 0.095, adjusted *P* = 0.32; TNF-α, *P* = 0.069, adjusted *P* = 0.31. *P* values for comparison between COV-ICU and sepsis: IL-1β, *P* = 0.36, adjusted *P* = 0.79; IL-1RA, *P* = 0.11, adjusted *P* = 0.32; IL-6, *P* = 0.45, adjusted *P* = 0.79; IL-8, *P* = 0.93, adjusted *P* = 0.99; IL-18, *P* = 0.0043, adjusted *P* = 0.078; TNF-α, *P* = 1, adjusted *P* = 1.

The primary focus of our analysis was on the levels of 6 cytokines associated with “cytokine storm”: IL-1β, IL-1RA, IL-6, IL-8, IL-18 and TNF-α. Levels of these cytokines did not significantly differ between the moderate COVID-19 (COV-noICU), severe COVID-19 (COV-ICU), ARDS, and sepsis groups (Figure 1; after multiple comparisons correction, *P* > 0.05). There was a trend toward higher levels of IL-1RA and IL-6 in the patients with severe COVID-19 as compared with those with moderate COVID-19, consistent with prior reports (1–3). There was also a trend toward higher IL-18 in the severe COVID-19 group compared with the sepsis group; however, this was not significant after multiple comparisons correction (adjusted *P* < 0.1).

In the more extended exploratory analysis of 70 additional cytokines, the 4 patient groups did not differ strongly in principal component analysis (Supplemental Figure 1; supplemental material available online with this article; https://doi.org/10.1172/jci.insight.140289DS1). There were small but statistically significant differences in the levels of IL-16, PDGF-BB, and thymic stromal lymphopoietin (TSLP) between the 4 groups (Supplemental Figure 2 and Supplemental Table 1). IL-16 was significantly lower in the moderate COVID-19 group compared with ARDS and sepsis. PDGF-BB was higher in moderate COVID-19 patients and ARDS patients compared with severe COVID-19. TSLP was significantly lower in both moderate and severe COVID-19 compared with ARDS and sepsis.

Among the other 67 cytokines, no significant differences were found between the 4 groups, which suggests that none of these cytokine levels were dramatically increased in patients with COVID-19 compared with critically ill patients with ARDS or sepsis (Supplemental Figure 3). The data from these additional cytokines are provided as a resource and, taken together, suggest that a “cytokine storm” in COVID-19 that is distinct from other critical illnesses (e.g., sepsis and ARDS) is unlikely.

## Discussion

Our primary goal was to directly compare levels of 6 inflammatory cytokines commonly associated with cytokine storm (IL-1β, IL-1RA, IL-6, IL-8, IL-18, and TNF-α) between severe COVID-19 patients and patients with ARDS or sepsis. Our findings are consistent with previous data demonstrating higher levels of inflammatory cytokines among COVID-19 patients with more severe disease. Importantly, however, our data suggest that inflammatory biomarkers in severe COVID-19 patients are not markedly elevated when directly compared with critically ill patients with ARDS or sepsis.

IL-6 levels were measured by the clinical lab as part of clinical care in 6 of the COVID-19 patients, including 4 with severe disease, though not at matched time points with the research blood collection. Levels ranged from < 6–31 pg/mL. These findings are consistent with recently reported IL-6 levels in severe COVID-19 patients (~10–40 pg/mL measured in clinical labs) (1–3) and are lower than levels reported in prior ARDS cohorts ( ~100–2000 pg/mL using ELISA) (10–12). Given the small number of patients in our cohort who had clinical measurements available, and the variation in collection times, we were unable to derive accurate concentrations based on median fluorescence intensity (MFI) for the remaining patients. Nonetheless, these data further support our findings that IL-6 levels in particular — while elevated above levels found in healthy subjects — are not markedly elevated in all severe COVID-19 patients compared with other critically ill patients.

This study calls into question the idea that “cytokine storm” is the major driver of morbidity and mortality in all severe COVID-19 patients. As Ritchie and Singanayagam have stated, it is equally possible that the higher levels of proinflammatory cytokines seen in severe COVID-19 reflect an increased burden of virus rather than “an inappropriate host response that requires correction” (13).

The most important limitation of this study is our small sample size. Even in our predetermined analysis of 6 cytokines strongly associated with cytokine storm, we lack power to detect minor differences between groups. The exploratory analyses of an additional 70 cytokines is similarly limited but is provided as a reference for the field. In addition, we do not have measurements of cytokines over time — only near the point of enrollment. Finally, as discussed above, we report cytokine levels by MFI per recommended Luminex analysis methods (14), precluding direct comparison of our values to previously published data that report cytokine levels in pg/mL.

Despite the above limitations, it is clear that, in this cohort, inflammatory cytokines are not dramatically higher in severe COVID-19 patients than in other critically ill patients. Broad testing of immunosuppressive therapies in unselected COVID-19 patients may therefore face the same fate as trials of immunosuppression in unselected ARDS and sepsis patients: inconclusive results despite decades of effort and the uncomfortable understanding that, while some patients may have been helped, others may have been harmed. Indeed, given the duration of mechanical ventilation and attendant high rates of nosocomial infection in severe COVID-19 patients, these therapies have potential for harm. Further research in larger cohorts is needed to characterize the spectrum of the immune response in COVID-19 and to identify which patients are most likely to benefit from (and least likely to be harmed by) immunomodulatory therapies.

## Methods

### Clinical cohorts.

Sequential inpatients with COVID-19 admitted to Stanford Hospital were approached for participation in the Stanford ICU biobank between mid-March and early April, 2020. Randomized clinical trials that required blood draws enrolled every other day during this period; these patients were excluded from biobank because of phlebotomy volume. COVID-19 patients were classified as “severe” if admitted to the ICU for respiratory failure and as “moderate” if they did not require ICU admission.

Twelve critically ill subjects with ARDS and 16 patients with sepsis who had been enrolled in the Stanford ICU biobank between 2015 and 2018 were selected for comparison. Briefly, the Stanford ICU Biobank recruits patients at risk for development of respiratory failure and ARDS admitted to Stanford Hospital as previously described (15). Subjects are eligible for enrollment when a decision to admit to ICU is made, either from the Emergency Department or the hospital wards, with goal enrollment in < 24 hours of ICU transfer. All patients were phenotyped for ARDS and sepsis by 2-physician consensus, based on the Berlin Criteria and Sepsis-2 criteria and using all available hospital clinical data, including history, physical exam, laboratory, and microbiologic data; invasive monitoring data; autopsy results; and physician summaries. Patients who met criteria for both ARDS and sepsis were classified as ARDS. Because the Stanford ICU is a major referral center for cancer, in order to assess cytokine response to infection in patients with a normal immune system (similar to the COVID-19 population), ARDS and sepsis patients were enriched for normal baseline immune system (e.g., no metastatic cancer, bone marrow transplant, or high dose steroids) in comparison with the Biobank as a whole.

### Sample collection and processing.

For patients with severe COVID-19, ARDS, and sepsis, biospecimens were collected within 24–48 hours of ICU admission in all but 1 patient, from whom the specimen was collected the following day (<72 hours). Among the moderate COVID-19 patients, biospecimens were collected within 24–48 hours of hospital admission in 4 of 6 patients, with the remaining 2 samples delayed, as these patients had been admitted to the hospital before our biobank received clearance to begin collecting specimens from COVID-19–positive patients. From each patient, 8–20 mL of whole blood was collected in EDTA tubes and was centrifuged at 300 *g* for 10 minutes at 25°C to separate blood cells and plasma. The plasma was removed and centrifuged again at 1200 *g* for 10 minutes 25°C to remove cell debris, and then aliquoted, frozen, and stored at –80°C. Plasma aliquots were thawed immediately before the Luminex assay.

### Luminex-EMD Millipore H76.

Luminex assays were performed at the Human Immune Monitoring Center at Stanford University. All samples were run in duplicate in a single plate per panel. Kits were purchased from EMD Millipore Corporation and used according to the manufacturer’s recommendations with modifications described as follows: H76 kits include 3 panels. Panel 1 is Milliplex HCYTMAG60PMX41BK with IL-18 and IL-22 added to generate a 43-plex assay. Panel 2 is Milliplex HCP2MAG62KPX23BK with MIG/CXCL9 added to generate a 24-plex assay. Panel 3 includes the Milliplex HSP1MAG-63K with Resistin, Leptin, and HGF added to generate a 9-plex assay. The setup of the assay was as recommended by the manufacturer. Briefly, samples were mixed with antibody-linked magnetic beads in a 96-well plate and incubated overnight at 4°C with shaking. Cold and room temperature incubation steps were performed on an orbital shaker at 500–600 rpm. Plates were washed twice with a wash buffer in a Biotek ELx405 washer. Following a 1-hour incubation at room temperature with biotinylated detection antibody, streptavidin-PE was added for 30 minutes with shaking. Plates were washed as above, and PBS was added to wells for reading in the Luminex L200 Instrument with a lower bound of 50 beads per sample per cytokine. Each sample was measured in duplicate. Custom Assay Chex control beads were purchased from Radix Biosolutions and were added to all wells, and their MFI was quantified and used to assess and normalize for nonspecific binding. The data supporting this publication are available at ImmPort (https://www.immport.org) under study accession SDY1645.

### Statistics.

All statistical analyses were performed using the open source statistical software R (https://www.r-project.org). Because we observed significant differences in CHEX4 MFI between the 3 groups tested, we corrected for CHEX4 nonspecific binding, keeping the clinical conditions (severe COVID-19 [COV-ICU], moderate COVID-19 [COV-noICU], ARDS and sepsis), age, and sex as covariates. Each sample was normalized according to methods used by the Human Immune Monitoring Center at Stanford University (16). Briefly, the MFI of each cytokine was corrected first for plate/batch/lot artifacts by linear mixed modeling; then, the average of technical replicates was log transformed. Afterward, the log-transformed average MFIs were corrected for nonspecific binding by local polynomial regression and repeated cross-validation, resulting in plate-detrended MFI (dpMFI) values.

Once normalized for nonspecific binding, levels of IL-1β, IL-1RA, IL-6, IL-8, IL-18, and TNF-α in severe COVID-19 patients (COV-ICU) were compared with levels in moderate COVID-19 patients (COV-noICU) and with levels in patients with the 2 other critical illnesses (ARDS and sepsis) using the Wilcoxon rank sum test corrected for multiple testing by the Benjamini-Hochberg method.

We performed principal component analysis (PCA) of the 70 additional cytokines. The PCA was computed by singular value decomposition of the dpMFI values of the 70 cytokines. The data ellipses were computed assuming a multivariate t-distribution with the ggfortify R package available on the CRAN repository. On these remaining 70 cytokines, Kruskal-Wallis tests were performed, and *P* values were corrected for multiple testing by the Benjamini-Hochberg method. On the cytokines with an adjusted *P* value lower than 0.05 with the Kruskal-Wallis test, we performed a Wilcoxon rank sum test as a post hoc test and corrected for multiple testing by the Benjamini-Hochberg method.

### Study approval.

This study was conducted according to the Declaration of Helsinki principles and was approved by the Stanford University Hospital IRB (protocol 28205). All patients or their surrogates gave written informed consent to participate in the Stanford ICU Biobank.

## Authors contributions

JGW, LJS, AMF, CAB, and AJR participated in project conception, design, and data interpretation. AR, JR, AA, TR, PMG, AS, JEL, and AJR contributed to patient enrollment, data acquisition, and sample processing. Luminex data acquisition was performed by YRH and HTM. Data analysis was performed by LJS and AMF, which were verified by SPH and CAB. The manuscript was drafted by JGW, LJS, AMF, CAB, and AJR, with critical revisions by AR, PMG, AS, YRH, SPH, and JEL. All authors approved the final version of this article. JGW, LJS, and AMF made equal and critical contributions; JGW is listed first for her role in the conception of the study; LJS and AMF played a critical role in data analysis and interpretation.

## Supplementary Material

Supplemental data

ICMJE disclosure forms

## Figures and Tables

**Table 1:**
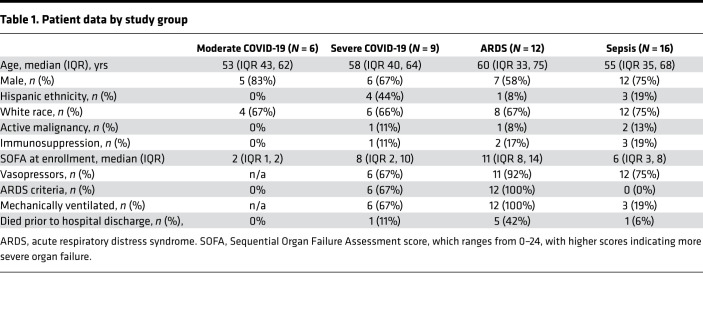
Patient data by study group

## Version 1 07/24/2020

In-Press Preview

## Version 2 09/03/2020

Electronic publication
