# Supplementary material for: Cytokine profile in plasma of severe COVID-19 does not differ from ARDS and sepsis
Source: JCI Insight. 2020 Sep 3;5(17):e140289. doi: 10.1172/jci.insight.140289 (PMC7526438; doi:10.1172/jci.insight.140289)
Supplement: Supplemental data [file jciinsight-5-140289-s109.pdf]

SUPPLEMENTARY MATERIAL

Cytokine profile in plasma of severe COVID-19 does not differ from ARDS and sepsis

Jennifer G. Wilson[1]*, Laura J. Simpson[2]*, Anne-Maud Ferreira[3]*, Arjun Rustagi[2], Jonasel Roque[2], Adijat Asuni[2], Thanmayi Ranganath[2], Philip M. Grant[2], Aruna Subramanian[2], Yael Rosenberg-Hasson[4], Holden T. Maecker[4], Susan P. Holmes[3], Joseph E. Levitt[2], Catherine A. Blish[2,4,5], Angela J. Rogers[2]

*These authors share first authorship.

[1]Department of Emergency Medicine, [2]Department of Medicine, and [4]Institute for Immunity, Transplantation, and Infection, Stanford University School of Medicine; [3]Department of Statistics, Stanford University, CA, USA; [5]Chan Zuckerberg Biohub, San Francisco, CA, USA

Corresponding author:
Angela J. Rogers, MD, MPH
300 Pasteur Dr A175, Stanford, CA 94305
650-724-5282
ajrogerss@stanford.edu

Contents:

Figure S1. Principal component analysis of 70 additional cytokines in moderate COVID-19, severe COVID-19, ARDS and sepsis.
Figure S2. Differentially expressed cytokines: IL-16, PDGF-BB, and TSLP.
Figure S3. Boxplots for the remaining 67 cytokines measured.
Table S1. Adjusted p-values for comparisons in PDGF-BB, IL-16, and TSLP.

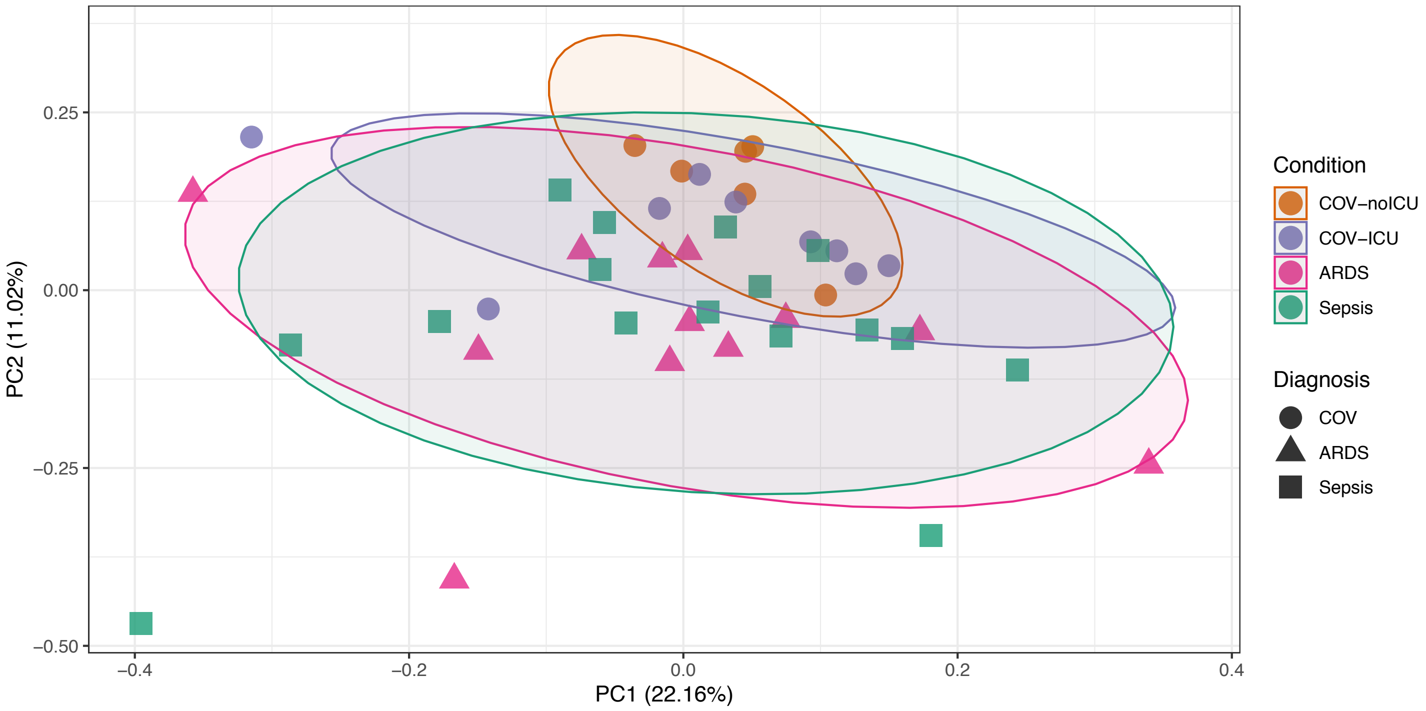

Supplemental Figure S1. Principal component analysis (PCA) of 70 additional cytokines in moderate COVID-19, severe COVID-19, ARDS and sepsis.
Moderate COVID-19 (COV-noICU, orange), n = 6. Severe COVID-19 (COV-ICU, purple), n = 9. ARDS (pink), n = 12. Sepsis (green), n = 16. PCA computed by singular value decomposition of the dpMFI values of the 70 cytokines, colored by condition. The first principal component (PC) explains 22.16% of the variability; the second PC explains 11.02% of the variability. The data ellipses were computed assuming a multivariate t-distribution with the ggfortify R package.

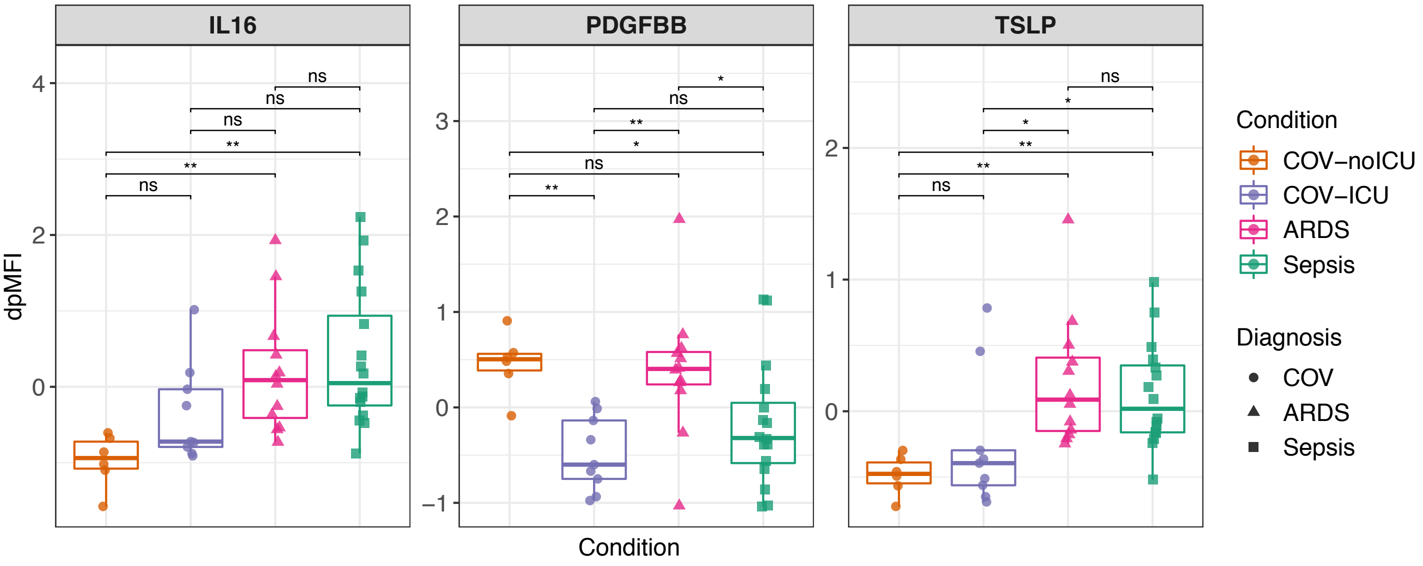

Supplemental Figure S2. Differentially expressed cytokines: IL-16, PDGF-BB, and TSLP. Box plots are shown for IL-16, PDGF-BB, and TSLP, which were the only 3 cytokines that had adjusted p-values < 0.05 by Kruskal-Wallis test with correction for multiple testing with the Benjamini-Hochberg method. Moderate COVID-19 (COV-noICU, orange), n = 6. Severe COVID-19 (COV-ICU, purple), n = 9. ARDS (pink), n = 12. Sepsis (green), n = 16. Adjusted p-values for specific comparisons shown in the plots were calculated by Wilcoxon rank sum tests with correction by the Benjamini-Hochberg correction method. The box plot visualizes the following summary statistics: the middle line represents the median; the lower hinge corresponds to the first quartile (25th); the upper hinge corresponds to the third quartile (75th); upper and lower whiskers extend from the hinge respectively to the largest value and smallest value or no further than 1.5 * interquartile range; data points beyond the whiskers are outliers.

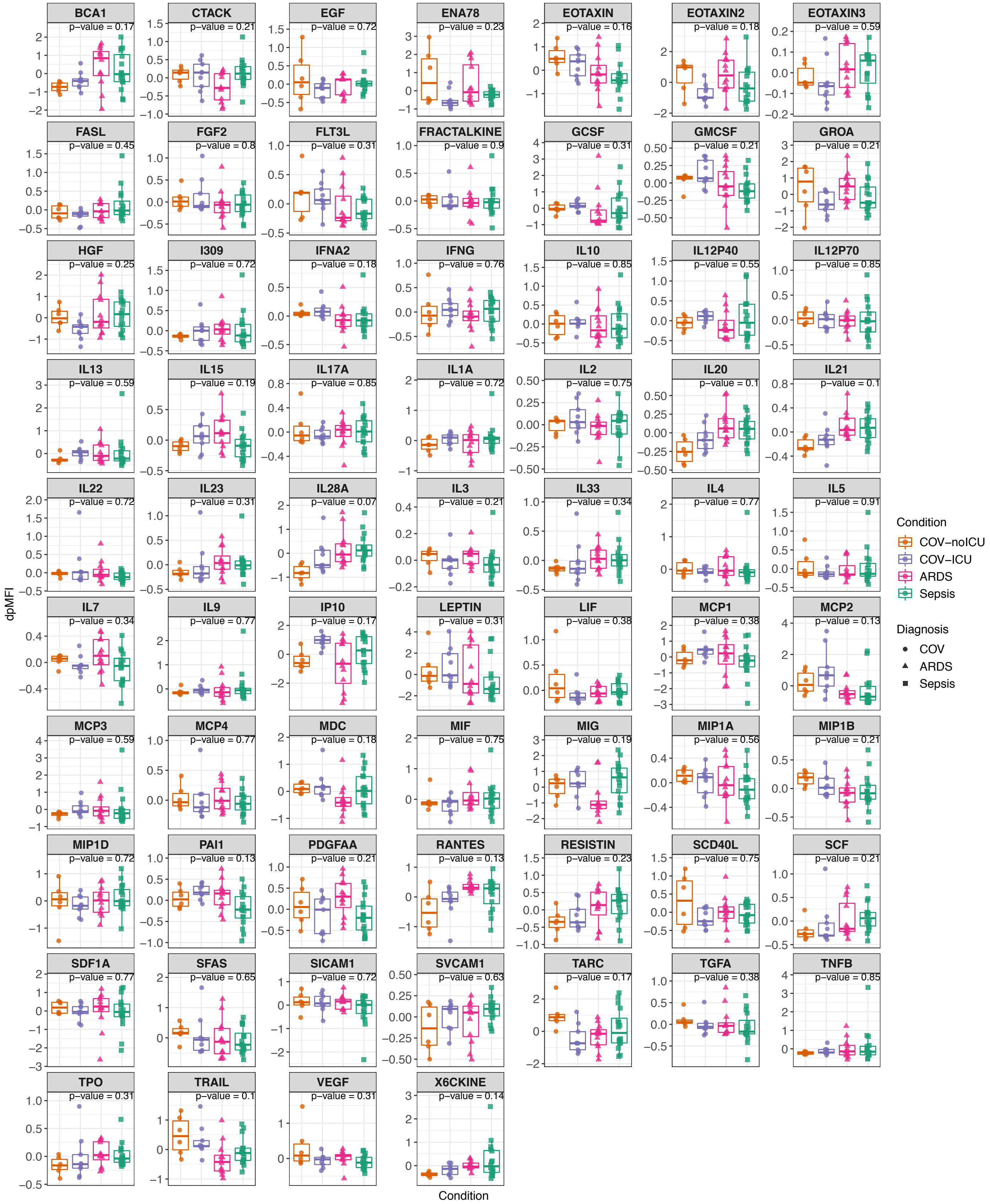

Supplemental Figure S3. Boxplots for the remaining 67 cytokines measured. Moderate COVID-19 (COV-noICU, orange), n = 6. Severe COVID-19 (COV-ICU, purple), n = 9. ARDS (pink), n = 12. Sepsis (green), n = 16. Adjusted p-values (shown in the top right of the plot for each cytokine) were all > 0.05 by Kruskal-Wallis test with correction for multiple testing with the Benjamini-Hochberg method. The box plot visualizes the following summary statistics: the middle line represents the median; the lower hinge corresponds to the first quartile (25th); the upper hinge corresponds to the third quartile (75th); upper and lower whiskers extend from the hinge respectively to the largest value and smallest value or no further than 1.5 * interquartile range; data points beyond the whiskers are outliers.

Cytokine	Group 1	Group 2	Significance	Adj p-value
PDGFBB	COV-noICU	COV-ICU	**	0.005
PDGFBB	COV-noICU	ARDS	ns	0.694
PDGFBB	COV-noICU	Sepsis	*	0.038
PDGFBB	COV-ICU	ARDS	**	0.005
PDGFBB	COV-ICU	Sepsis	ns	0.423
PDGFBB	ARDS	Sepsis	*	0.039
IL16	COV-noICU	COV-ICU	ns	0.122
IL16	COV-noICU	ARDS	**	0.002
IL16	COV-noICU	Sepsis	**	0.002
IL16	COV-ICU	ARDS	ns	0.122
IL16	COV-ICU	Sepsis	ns	0.070
IL16	ARDS	Sepsis	ns	0.703
TSLP	COV-noICU	COV-ICU	ns	0.634
TSLP	COV-noICU	ARDS	**	0.002
TSLP	COV-noICU	Sepsis	**	0.002
TSLP	COV-ICU	ARDS	*	0.038
TSLP	COV-ICU	Sepsis	*	0.042
TSLP	ARDS	Sepsis	ns	0.767

Supplemental Table S1. Adjusted p-values for comparisons in PDGF-BB, IL-16, and TSLP. Moderate COVID-19 (COV-noICU), n = 6. Severe COVID-19 (COV-ICU), n = 9. ARDS, n = 12. Sepsis, n = 16. Adjusted p-values for specific comparisons shown in the plots in Figure S2 were calculated by Wilcoxon rank sum tests with correction by the Benjamini-Hochberg correction method.
